# Genome-wide dynamic network analysis reveals the potential genes for MeJA-induced growth-to-defense transition

**DOI:** 10.1186/s12870-021-03185-1

**Published:** 2021-10-06

**Authors:** Tengfei Wang, Xiujun Zhang

**Affiliations:** 1 CAS Key Laboratory of Plant Germplasm Enhancement and Specialty Agriculture, Wuhan Botanical Garden, Chinese Academy of Sciences, 430074 Wuhan, China; 2 Center of Economic Botany, Core Botanical Gardens, Chinese Academy of Sciences, 430074 Wuhan, China; 3 University of Chinese Academy of Sciences, 100049 Beijing, China

**Keywords:** Methyl jasmonate (MeJA), Growth-to-defense transition, Plant resistance, Dynamic network biomarker (DNB), Tipping point

## Abstract

**Background:**

Methyl jasmonate (MeJA), which has been identified as a lipid-derived stress hormone, mediates plant resistance to biotic/abiotic stress. Understanding MeJA-induced plant defense provides insight into how they responding to environmental stimuli.

**Result:**

In this work, the dynamic network analysis method was used to quantitatively identify the tipping point of growth-to-defense transition and detect the associated genes. As a result, 146 genes were detected as dynamic network biomarker (DNB) members and the critical defense transition was identified based on dense time-series RNA-seq data of MeJA-treated *Arabidopsis thaliana*. The GO functional analysis showed that these DNB genes were significantly enriched in defense terms. The network analysis between DNB genes and differentially expressed genes showed that the hub genes including SYP121, SYP122, WRKY33 and MPK11 play a vital role in plant growth-to-defense transition.

**Conclusions:**

Based on the dynamic network analysis of MeJA-induced plant resistance, we provide an important guideline for understanding the growth-to-defense transition of plants’ response to environment stimuli. This study also provides a database with the key genes of plant defense induced by MeJA.

**Supplementary Information:**

The online version contains supplementary material available at 10.1186/s12870-021-03185-1.

## Introduction

In response to changing environments, plants have to manage their resources to reach a balance between growth and defense in order to survive and reproduce [1, 2]. Rapid growth and accumulation of nutrients help plants compete for more resources. While an effective and inducible defense system at the cost of resources has evolved in plants to resist biotic/abiotic stress [3, 4]. The correct and effective transition from growth to defense is crucial for plants’ survival and wellbeing [5, 6]. Several studies have correlated some phytohormones with the regulation of plant defense and identified multiple factors as the participants of the pathway [7–9]. Revealing the basic mechanism of hormone-induced growth-to-defense transition is essential to elucidate how plants respond to environmental stimuli [10, 11] and carry important implications to ecological and agricultural improvement [12, 13].

Methyl jasmonate (MeJA) and its free-acid jasmonic acid (JA) were well-recognized as lipid-derived stress hormones [14, 15]. JAs-mediated growth-to-defense tradeoffs regulate plant defense against necrotrophic pathogens and insect herbivores [16, 17]. In previous studies, many genes of the JA signaling pathway were identified as participants of plant defense [18–20]. By the crosstalk with other phytohormones, JAs take part in the defense against abiotic stresses and biotic stresses [21–23]. These results indicated that JAs-induced growth-to-defense transition plays a crucial role in plant defense against the changing environments [24, 25].

To understand the molecular mechanism of plants’ defense induced by JAs, some genetic, molecular, and physiological approaches have been used and some results were achieved. The core JA signaling pathway was discovered as a co-receptor complex consisting of the F-box protein CORONATINE INESENSTIVE 1 (COI1) and the JASMONATE ZIM DOMAIN (JAZ) family of transcription repressors. The COI1–JAZ complex perceives jasmonoyl-l-isoleucine (JA-Ile), leading to poly-ubiquitylation of JAZ and its subsequent degradation. The degradation of JAZ relieves the repression of JAZ-interacting transcription factors, permitting the expression of related genes and execution of physiological responses, with multiple feedback loops to ensure timely termination of these responses [18, 26].

Network-based analysis methods have been developed to identify candidate genes and elucidate the molecular mechanisms of complex biological processes at a system level [27, 28]. Among the network inference methods, mutual information-based methods enable accurate quantification of associations in gene regulatory networks [29, 30]. The weighted correlation network analysis (WGCNA) has been widely used to detect gene modules that were related to some special traits [31]. In recent studies, network-based analysis methods have also been used to investigate the mechanism of growth-defense tradeoffs under stress or defense signals in plants [32, 33].

In recent years, the phase transition of disease development has been studied, such as the pre-disease state of diabetes mellitus and the critical transition of breast cancer [34, 35]. For plants, our previous work has studied the state transition of flowering and some genes were identified as the controllers of flowering [36]. The transition of complex system is usually non-smooth or abrupt in many biological processes [37]. The plant system in growth or defense state is stable and highly resilient. Plant usually survives by balancing growth and defense, i.e. it accumulates nutrients by former whereas costs resources by latter [1].

During the balance between plant growth and defense, the tipping point is the critical transition point of the transition of the system from growth to defense. The tipping point is not only a time point but also a response process of the system. At the tipping point, the expressions of the related genes fluctuate drastically. The unstable state also leads to increased fluctuation in the observed transcript expression level. In transition state, the violent fluctuations within the plant system include the transmission of defense signals, the redistribution of metabolic pathways, and the reallocation of resources. Identifying the critical state or tipping point of growth-to-defense transition is important to understand the systemic defense in plants. The system at the tipping point is fragile and susceptible to change. Therefore, the tipping point-associated genes are vital to the defense system [1, 2].

The time-series high-throughput sequencing data provide an unprecedented opportunity to study the mechanism of plant systemic resistance at the genome-wide scale [38, 39]. Here, a dynamic network analysis method called DNB, which was designed to quantitatively identify the tipping point of a drastic system transition, can theoretically identify a group of highly correlated and strongly fluctuated genes [34, 37].

In this work, we applied the DNB method to detect the critical point of plant grow-to-defense transition and identify plant resistance-associated genes. As a result, 146 genes were detected as DNB members and the 11th time (8 h) period was identified as a critical transition point based on time-series high-throughput RNA-seq data of MeJA-treated *Arabidopsis thaliana*. Among the DNB genes, many of them have been proved to be widely involved in plant defense. The GO functional enrichment analysis showed that these DNB genes were significantly enriched in defense terms. The STRING network with 287 nodes and 807 edges confirmed the close association between the DNB genes and the differentially expressed genes. The network analysis also showed that the hub genes including SYP121, SYP122, WRKY33, MPK11 and at5g22920 play a vital role in plant growth-to-defense transition. Based on the dynamic network analysis of MeJA-induced plant resistance, we provide an important guideline in understanding the growth-defense transition of plant responding to environment stimuli. We also provide a database with the key genes of plant defense induced by MeJA.

## Results

### Composite index for detecting the tipping point of growth-defense transition

**Fig. 1:**
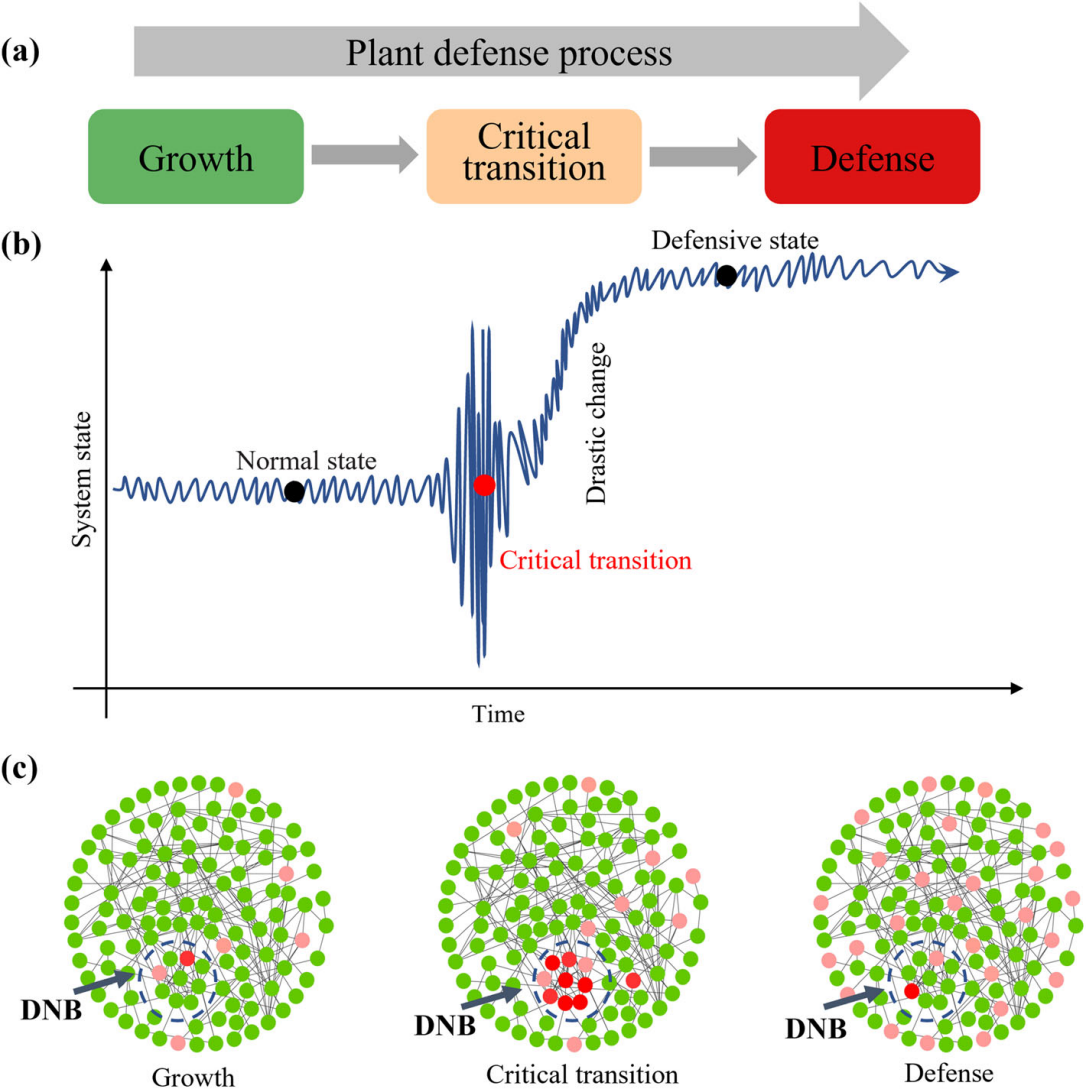
The schematic diagram for the identification of the critical point of plant growth-defense transition by dynamic network analysis. **a** The plant defense process from rapid growth to depletion of resources for defense can be divided into three states: growth state, critical transition state and defense state. **b** The system in growth or defense state is stable and resilient. In transition stage, the violent fluctuations within the plant system include the transmission of defense signals, the dramatic fluctuations in gene expression, and the redistribution of metabolic pathways. **c** In the critical transition state, the deviations of DNB members increase sharply and the correlations between DNB members increase obviously

The transition of complex system is usually non-smooth or abrupt in many biological processes. Such a transition changes the state of the biological system qualitatively and thus plays key roles in biological processes. The process of plant from rapid growth and accumulation of nutrients to depletion of resources for defense can be divided into three stages, i.e. growth state, critical transition state and defense state (Fig. 1a).

The system in growth or defense state is stable and highly resilient. In transition state, the violent fluctuations within the plant system include the transmission of defense signals, the dramatic fluctuations in gene expression, and the redistribution of metabolic pathways (Fig. 1b). To identify the tipping point of defense transition and understand the molecular mechanism of growth-defense tradeoffs, the dynamic network analysis method (DNB, dynamic network biomarkers) was used. The DNB method can detect critical transition state from the measured data based on nonlinear dynamic theory, which has been theoretically and numerically proved. A system is near to the critical transition state or tipping point if there is a dominant group of molecules or genes satisfying the following three criteria from the observed data: (1) Standard deviations of genes in this dominant group (denoted by *SDi*) increased markedly; (2) Pearson correlation coefficients between genes in this dominant group (denoted by *PCCi*) increased significantly; (3) Pearson correlation coefficients between genes in the group and genes outside of the group (denoted by *PCCo*) decreased significantly. The following composite index (CI) approximately considering all three criteria can be used as the numerical signal of the DNB method:

$$CI= SDi\ast PCCi/ PCCo,$$

where *SDi* is the average standard deviation of all genes in the dominant group, *PCCi* is the average PCC of all gene-pairs in the dominant group (absolute value), and *PCCo* is the average PCC of gene-pairs between the dominant group and others (absolute value). The definition of CI shows that there are a group of genes fluctuating strongly in the critical transition state. The group members are the dynamic network biomarkers of the critical transition. When *CI* reaches a peak or increases markedly during the periods, the biological system is at the critical transition state or tipping point (Fig. 1c).

In contrast to the traditional differential expression analysis, the DNB method considered both molecular fluctuation information (dynamic information) and network information (correlation information among genes).

### Dynamic network analysis of MeJA-induced plant defense

**Fig. 2:**
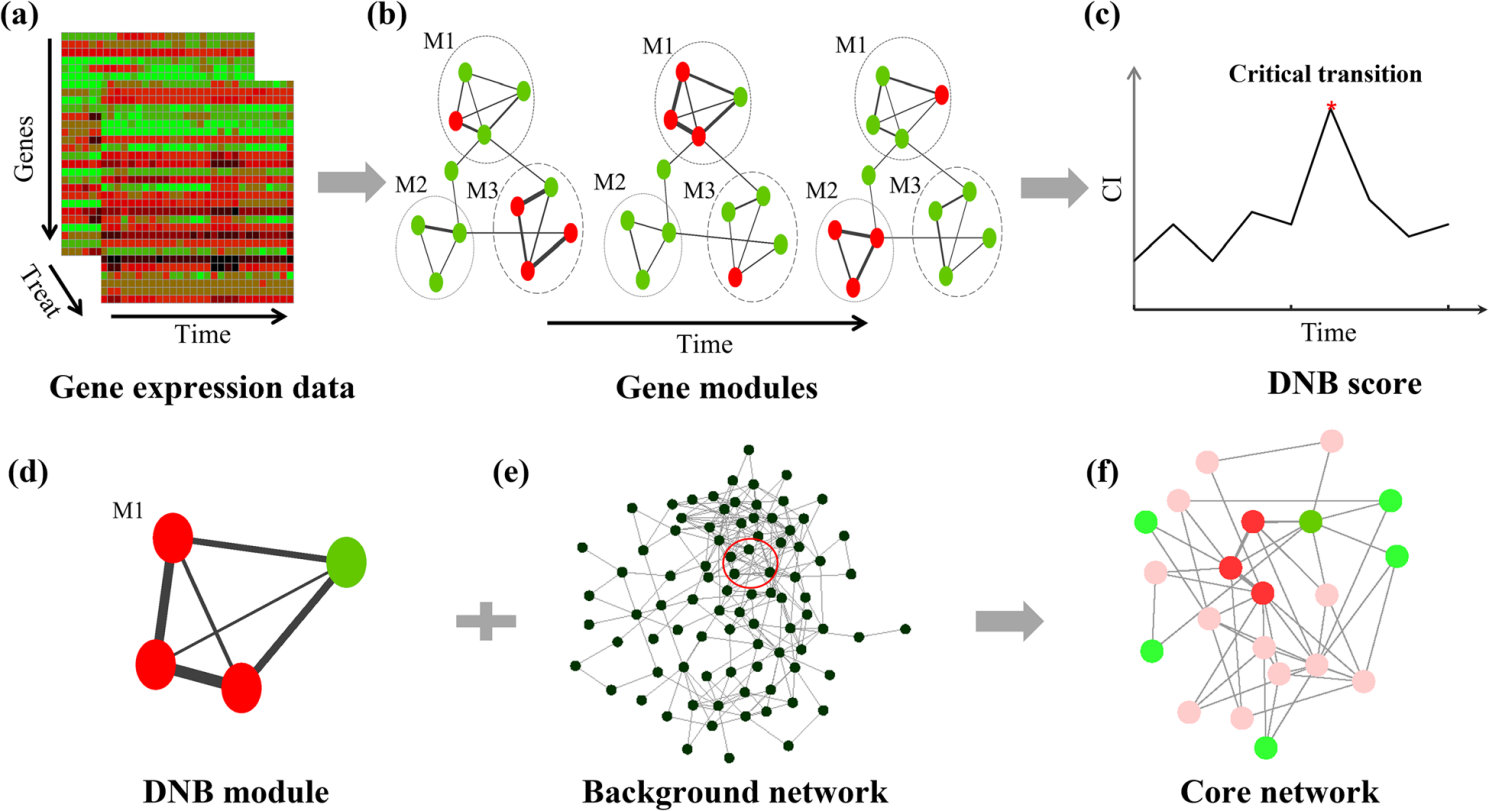
Flowchart of dynamic network analysis of MeJA-induced plant defense. **a** Control and MeJA-treated time-series gene expression data were normalized. **b** Gene modules (e.g., M1, M2, and M3) were constructed based on Pearson correlation coefficients between genes. Genes with standard deviation (SD) increased sharply are shown in red, others are in green. **c** CI scores were calculated to quantify DNB module. The time point with the highest CI was detected as the tipping point of transition (critical transition state), marked in red. **d** DNB modules were inferred from the candidate of gene modules. The module of genes with the highest CI_max was recognized as DNB module. **e** Protein-protein interactions (PPIs) from STRING network. The high confidence of interactions was used as background network for DNB genes. **f** Core network for plant defense were constructed based on DNB genes and background network

DNB is a method to identify gene module for the transition. In a DNB module, the standard deviations of genes increase markedly, the correlations of genes increase significantly while the correlations between genes in and out of the module decrease significantly. The procedure for the dynamic network analysis was described in Fig. 2. The time-series high-throughput sequencing data was aligned and assembled to obtain gene expression matrix for further dynamic network analysis (Fig. 2a).

The dynamic network analysis of plant growth-defense transition was performed with the following steps. Firstly, hierarchical clustering was implemented on the gene expression data to obtain candidate gene modules for each time point (Fig. 2a, b). Secondly, the CIs of candidate modules were calculated and the maximum CI (noted as CI_max) was selected for each time point. The selected CI_maxs for all the time points were compared to detect the tipping (critical transition) point. The time point with highest CI_max was considered as the critical transition point (Fig. 2b, c). Thirdly, at the detected critical transition point, the module with the highest CI_max was recognized as DNB module (Fig. 2d). Lastly, the *Arabidopsis thaliana* protein-protein interactions (PPIs) in STRING database were used as background network (Fig. 2e). The core network for plant growth-defense transition was constructed from DEGs directly interacted with DNB genes in background network (Fig. 2d, f).

### Tipping point of plant defense identified by DNB

**Fig. 3:**
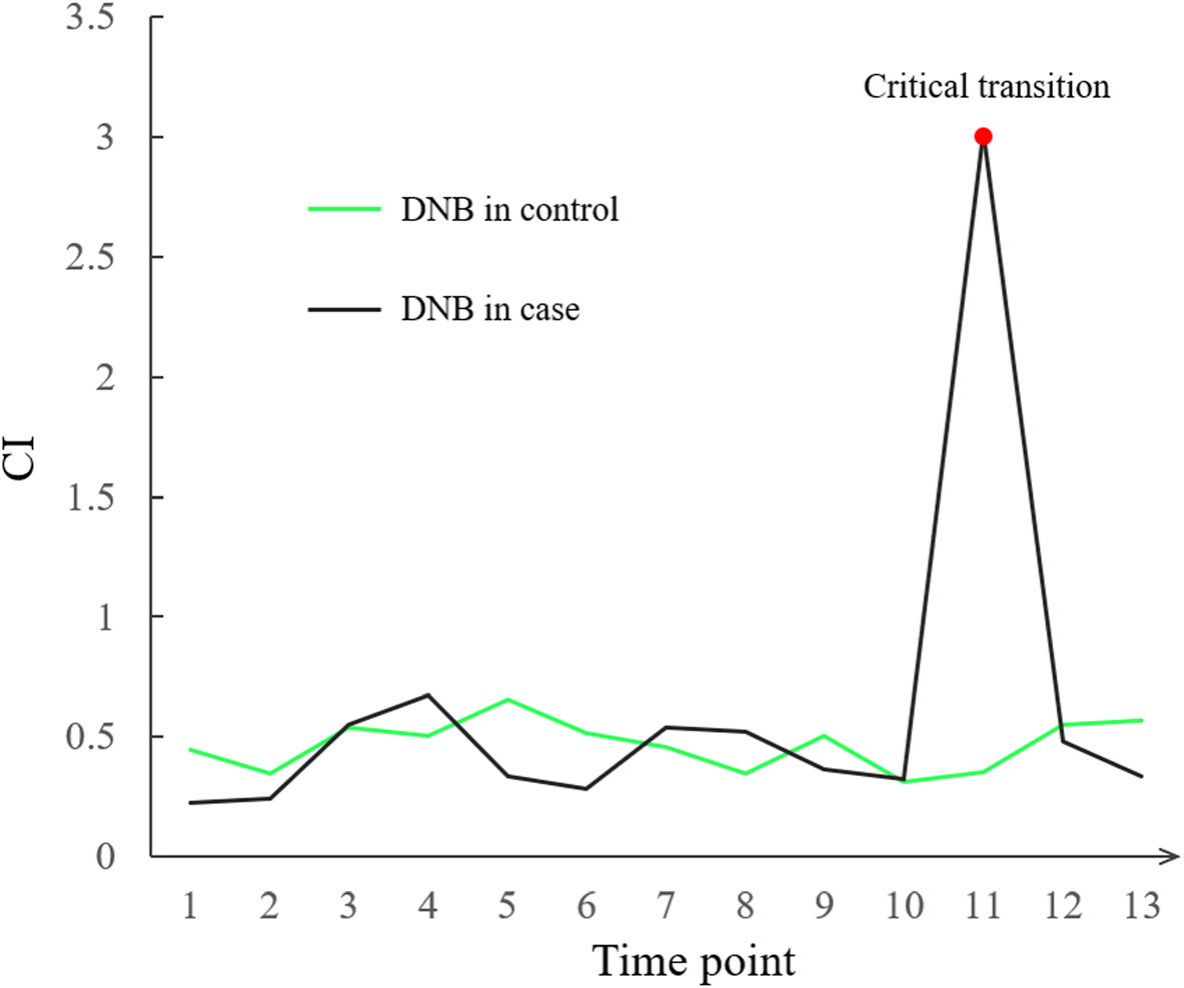
The CI of DNB module in different periods. The CI of DNB module was marked in black in data from case samples (MeJA treated) and was marked in green in data from control samples (MOCK treated). The highest CI score for DNB module marked as a red dot at point 11

The time-series RNA-seq dataset of MeJA-treated *Arabidopsis thaliana* at 13 time points was analyzed to detect the critical transition of plant growth-to-defense and identify the associated genes. As is shown in Fig. 3, the 11th time point (8 h after MeJA treatment) with the highest CI_max value (3.03) in all modules was identified as the critical transition state of plant defense and marked in red.

By analyzing the CIs of DNB modules, we found that CI reached highest at the tipping point. At the critical transition state, the average standard deviation (SD) of DNB members reached the maximum, the correlation increased significantly and the correlation outside the DNB module was lowest (Supplementary figure S1). The dynamics of SD showed that the expression of DNB genes was highly fluctuating at the tipping point. The correlation analysis of genes in and out of the DNB module showed that the DNB genes were highly correlated with the genes in the module while weakly correlated with the genes outside of the module (Supplementary Figure S1). This result showed that the emergence of such a cluster of genes approved it a signal of developmental state transition.

To verify the biological and statistical significance of the identified DNB module, the bootstrap analysis was performed. The result showed that it was significant for the CIs of DNB modules compared with the CIs of random gene modules (Supplementary Figure S2).

### Identified DNB genes are widely involved in plant defense

As another result of the dynamic network analysis, a DNB module with 146 genes was identified as the key genes for plant growth-to-defense transition. With the gene function annotation, we found that that the genes identified by DNB were widely involved in the initiation of plant defense and some of them played key roles in the regulation of plant defense. For instance, as a central metabolic regulator, transcription factor bZIP63 is essential for stress responses [40, 41]. Another ZIP transcription factor HAT22 regulates a set of genes implicated in stress responses and restricts new shoot growth [42]. Gene MYB3R5 with repressor function restrict the expression of gene with mitotic functions to inhibit plant growth [43]. Gene TOPP9 encodes Type One Protein Phosphatase that acts as a nucleocytoplasmic negative regulator of tip growth and affects the mitogen-activated protein kinases (MAPKs)-mediated downstream defense pathway [44]. It is a remarkable note that the DNB method can identify genes that play a key role in function but not show significant differential expression.

The differential expression analysis of DNB genes was performed between the critical transition points (8 h after MeJA treated) and the previous point (7 h after MeJA treated). Table 1 showed the functional description and expression changes of the DNB genes which were crucial for defense transition. The genes with fold change (FC) > 1.5 and false discovery rate (FDR) < 0.05 were selected as DEGs. Moreover, all the DNB genes with gene description were shown in Supplementary Table S1.

**Table. 1:** Functional description and expression changes of the DNB genes which crucial for defense transition

DNB Genes	Fold Change	Gene Description
**Transcription factor**		
bZIP63	25.81 ^b^	BASIC LEUCINE ZIPPER protein which regulates the circadian oscillator gene PRR7 to change the circadian phase in response to sugars.
HAT22	2.34 ^a^	Encodes homeobox protein HAT22, member of the HD-Zip II family. The mRNA is cell-to-cell mobile.
ERF043	3.33 ^a^	Encodes a member of the DREB subfamily A-4 of ERF/AP2 transcription factor family.
CRF6	4.15 ^a^	CRF6 encodes one of the six cytokinin response factors.
BPC1	2.83	BASIC PENTACYSTEINE1 (BPC1) is a regulator of the homeotic Arabidopsis thaliana gene SEEDSTICK (STK).
MYB3R-5	1.65	Encodes a putative c-MYB-like transcription factor of the MYB3R factor gene family.
AT5G26865	1.27	AGAMOUS-like MADS-box protein
AT3G58630	1.08	sequence-specific DNA binding transcription factor
**Response to stress**		
TCH3	5.68 ^b^	Encodes a calmodulin-like protein, with six potential calcium binding domains. Expression may also be developmentally controlled.
ERD15	3.77 ^b^	Encodes hydrophilic protein lacking Cys residues that is expressed in response to drought stress, light stress and treatment with plant-growth-promoting rhizobacteria.
TMAC2	21.70 ^b^	Encodes a protein that acts in the nucleus and is an important negative regulator of ABA and salt stress responses.
AGB1	1.78 ^b^	Encodes the heterotrimeric G-protein beta subunit and is involved in organ shape. A significant fraction of the protein is found in the ER.
AT1G03610	2.95 ^b^	plant/protein (DUF789)
CINV1	1.85 ^b^	CINV1 is an alkaline/neutral invertase that breaks sucrose down into fructose and glucose (GH100).
PP2-A13	4.52 ^b^	phloem protein 2-A13
AT4G26750	1.87 ^b^	Encodes a protein involved in mediating plant responses to pathogenesis.
PLIP2	9.32 ^b^	PLIP2 is a glycerolipid A1 lipase with substrate preference for monogalactosyldiacylglycerol. Expression is induced by ABA.
SAP2	4.65 ^b^	Eukaryotic aspartyl protease family protein
KPI-1	2.11 ^a^	Encodes a Kazal-type serine proteinase inhibitor that is highly expressed in seedlings and flowers.
ACBP3	5.07 ^a^	acyl-CoA-binding protein ACBP3. Shows up-regulation of many biotic and abiotic stress related genes.
SYP121	2.19	Encodes a syntaxin localized at the plasma membrane. SYR121/PEN1 is a member of the SNARE superfamily and functions in positioning anchoring of the KAT1 K + channel protein at the plasma membrane.
ERD14	1.52	Encodes a dehydrin protein whose expression is induced early on in response to dehydration stress.
MBL1	6.81	curculin-like (mannose-binding) lectin family protein, low similarity to ser/thr protein kinase from Zea mays; contains Pfam lectin (probable mannose binding) domain PF01453 but not the protein kinase domain of the Z. mays protein.
AT1G19310	1.51	RING/U-box superfamily protein
UGT76C2	1.78	Encodes a cytokinin N-glucosyltransferase that is involved in cytokinin homeostasis and cytokinin. Expression is induced by ABA, mannitol and drought stress.
SAG21	3.18	Encodes AtLEA5 (late embryogenesis abundant like protein). Also known as SENESCENCE-ASSOCIATED GENE 21 (SAG21). Elevated in response to various stresses.
SGT1A	1.39	Closely related to SGT1B, may function in SCF(TIR1) mediated protein degradation. AtSGT1a and AtSGT1b are functionally redundant in the resistance to pathogenes.
ASAP1	5.62	NSE5 subunit of the SMC5/6 complex.
EAP3	1.80	EAP3 is a cytolsolic BTB/POZ-domain protein involved in trafficking of PEN3.
RMA1	1.98	RMA1 encodes a novel 28 kDa protein with a RING finger motif and a C-terminal membrane-anchoring domain that is involved in the secretory pathway.
SYP42	1.63	Encodes a member of SYP4 Gene Family that is a plant ortholog of the Tlg2/syntaxin16 Qa-SNARE. Together with SYP43, it regulates the secretory and vacuolar transport pathways in the post-Golgi network.
NTAQ1	0.77	Controls the expression of specific defence-response genes, activates the synthesis pathway for the phytoalexin camalexin and influences basal resistance to Pseudomonas syringae pv tomato (Pst).
**Negative regulation of growth**		
TOPP9	4.35 ^b^	Encodes a Type One Protein Phosphatase that acts as a nucleocytoplasmic negative regulator of tip growth.
CDPK2	7.21 ^b^	Encodes a Ca(2+)-dependent, calmodulin-independent protein kinase that is rapidly induced by drought and high-salt stress. Positive regulator of ABA signaling.

^a^ indicates significance at 0.05 level ^b^ indicates significance at the 0.01 level

### Function analysis for DNB genes

**Fig. 4:**
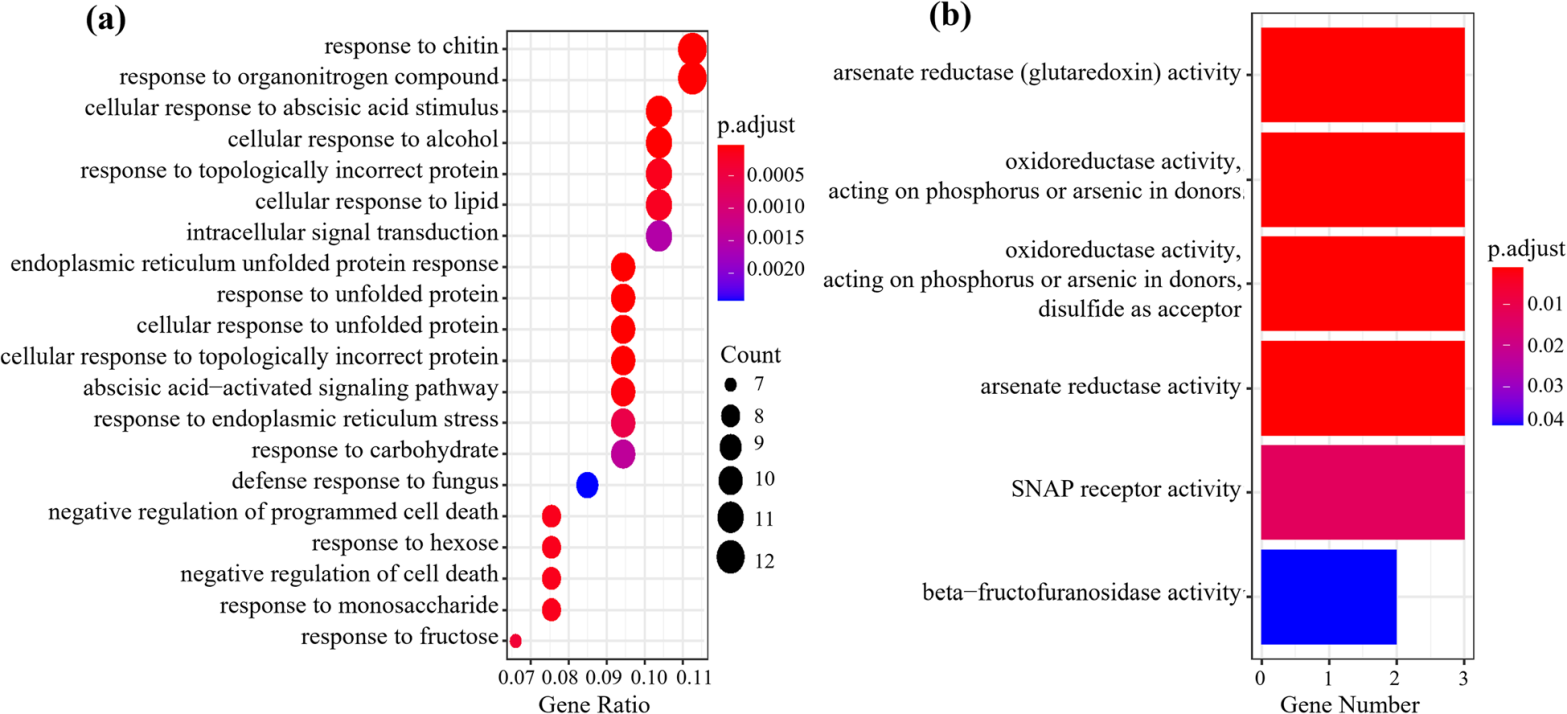
Function enrichment analysis of DNB genes. **a** The dotplot of biological process category enrichment. **b** The barplot of molecular function category enrichment

The Gene Ontology (GO) term enrichment analysis was implemented to classify the function of DNB genes at the critical transition of *Arabidopsis thaliana* induced by MeJA. In the biological process category, ‘response to chitin’ and ‘response to other organonitrogen compound’ were most common GO terms for the DNB genes. Some other significantly enriched GO terms include ‘response to unfolded protein’, ‘regulation of innate immune response’, and so on (Fig. 4a). In the molecular function category, these GO terms were enriched in DNB genes, such as ‘arsenate reductase (glutaredoxin) activity’, ‘oxidoreductase activity’, ‘acting on phosphorus or arsenic in donors’, ‘disulfide as acceptor’, ‘SNAP receptor activity’, and ‘beta-fructofuranosidase activity’ (Fig. 4b). None GO term was enriched in the cellular component category. All the enriched GO terms of DNB genes in the two GO categories (biological process and molecular function) were listed in Supplementary Table S2.

The Kyoto Encyclopedia of Genes and Genomes (KEGG) metabolic pathway analysis was implemented for checking the additional functional information of these identified genes [45]. These DNB genes were assigned to the reference pathway in KEGG to further check the biological function. As a result, 30 genes were mapped to about 26 pathways. The ‘plant-pathogen interaction’ pathway is the most common among these pathways. Besides, genes ARR4 and PYL5 were found to be involved in ‘plant hormone signal transduction’, genes PGL1 and GDH2 were found to be involved in ‘carbon metabolism’ pathway, and genes SYP121 and SYP42 were found to be involved in ‘SNARE interactions in vesicular transport’ pathway. The DNB genes assigned KEGG pathways were shown in Supplementary Table S3. The GO and KEGG analyses revealed that DNB genes were closely related to plant defense and signaling pathway.

### Core network of DNB genes and differentially expressed genes

**Fig. 5:**
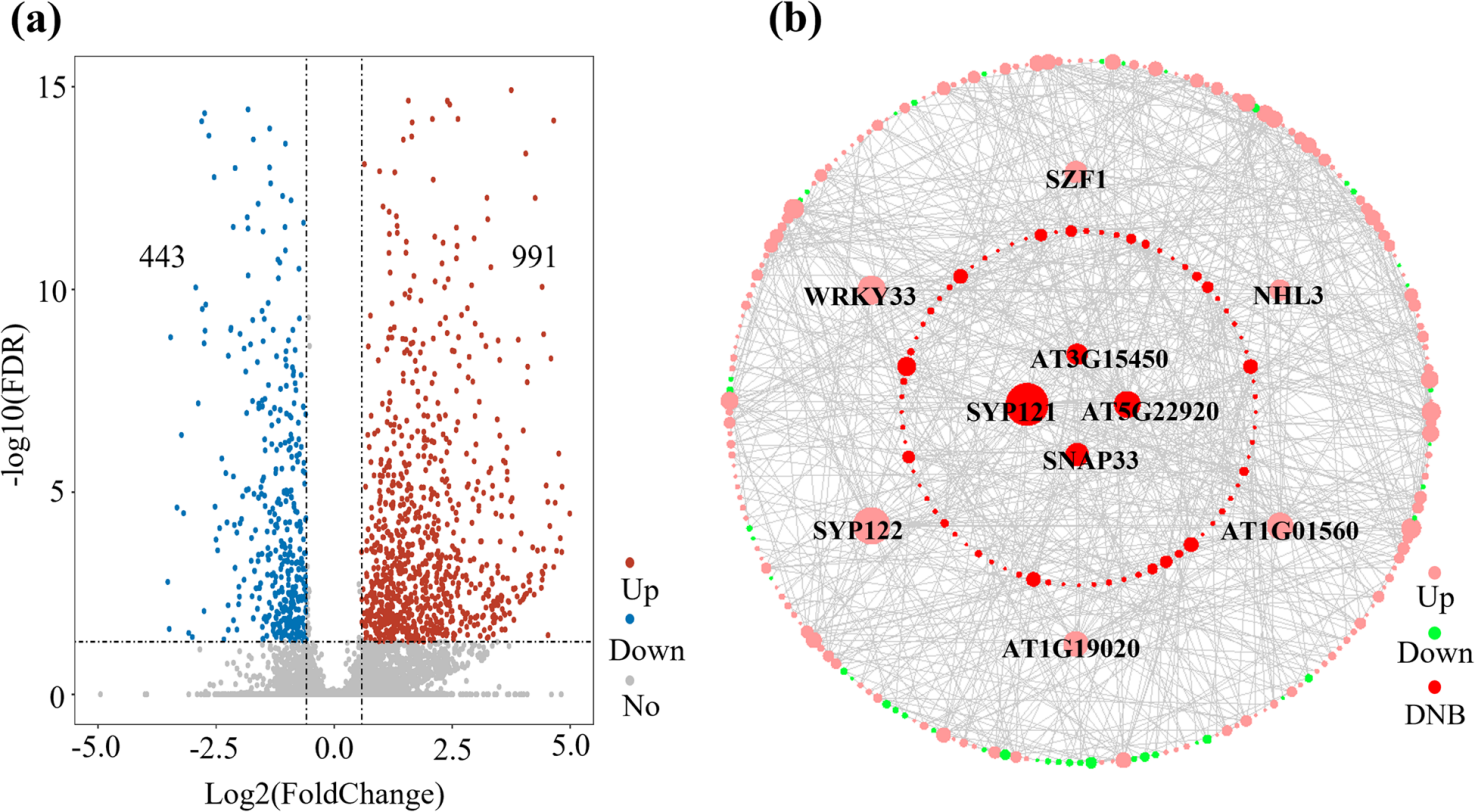
The core network of DNB genes and DEG genes. **a** Volcano plot to display differential expressed genes between defense tipping point and previous time. **b** The core network between DNB genes and DEG genes. Nodes were sized according to connection degree. DNB genes were marked in red, up-regulated genes were marked in pink, and down-regulated genes were marked in green

To discover the associations between dynamic network biomarker (DNB) genes and differentially expressed genes (DEGs), the differential gene expression analysis was performed for defense tipping point and previous time. The result showed that 1434 genes (Supplementary Table S4) were differentially expressed between the defense tipping point and previous time point. Among these genes, 991 genes were up-regulated and 443 genes were down-regulated (Fig. 5a). Subsequently, a core network was constructed consisting of 287 nodes with 708 edges between DNB genes and DEGs based on the STRING network (Fig. 5). In this network, the red nodes represented DNB genes, green nodes represented down-regulated DEGs, and pink nodes represented the up-regulated DEGs. The network of DNB genes and DEGs was shown in Supplementary Table S5.

In addition, we analyzed the function of hub genes of the core network in plant growth-to-defense transition. The result showed that 7 out of the top 10 hub genes including SYP121, SYP122, WRKY33, NHL3, TZF11, and AT1G19020 play a key role in the regulation of plant resistance. The functional descriptions of the top 10 hub genes were shown in Supplementary Table S6.

## Discussion

The growth-defense transition is the form of plant responding to (a)biotic stress or defending signals. This method is an effective approach to study crop improvement by identifying the genes that control the growth-defense transition [46–48]. With the function of rewiring central metabolism to promote defense and inhibit growth, JAs play a key role in growth and defense trade-offs [24, 26, 49]. Some genes related to growth-defense transition have been found in the JAs pathway [48, 50].

The high-resolution time-series RNA-seq data provide the opportunity to identify the critical state or tipping point of plant growth-defense transition [51]. At the tipping point, the JA response genes in format of a module fluctuate drastically in expression. For example, the expression of these genes decreased in plant growth phase while increased in defense phase. Subsequently, the plant minimizes growth and initiated defense. These genes were taken as the key factors for the growth-defense transition [52]. In our opinion, the tipping point is the transition state of plant growth-defense transitions in theory. For the result of data analysis, the detected tipping point was just the nearest one to the transition state among all the time points measured for the time-intensive data. The methods for identifying key genes that involve in the JA gene regulatory network are important for understanding of plant growth-defense transitions [38]. To understand the mechanism of plant growth-defense transitions, the special methods which can accurately model JA defend response should be developed [32].

To investigate the JA gene regulatory network, high-resolution time series RNA-seq for MeJA-treated *Arabidopsis thaliana* were generated [51]. With computational analysis, this study provided insight into the dynamics and architecture of the JA gene regulatory network. Several TFs including MYB59 and bHLH27 were uncovered as early network components with a role in pathogen and insect resistance. What’s more, this study also provided an information-rich resources to investigate JA signaling components in plant growth and defense [51].

Here, we provide a novel insight to identify the critical point and key genes of plant growth and defense transition response to JA. The DNB method identified the appearance of a group of genes with strongly collective fluctuations from the measured gene expression data to determine the critical transition of growth-to-defense in plants. Furthermore, the group members were predicted as the dynamic biomarkers for this critical transition. Based on the time-series high-throughput RNA-seq data of MeJA-treated *Arabidopsis thaliana*, we identified the critical growth-to-defense transition state and key genes by applying the non-modeled DNB approach.

The trade-offs of plant growth-defense have been studied and discussed a lot. These studies showed that the critical transition of growth-to-defense is key for plant defense. In the result of our study, the time point 8 h (8 h after MeJA treatment) was identified as the starting point of defense from minimizing growth and 146 genes were selected as DNB members. These genes detected by DNB extensively participate in the redistribution of metabolism which was involved in plant defense. For instance, activated by protein kinase complexes SnRK1, bZIP63 was considered as the central metabolic regulator essential for stress responses [40, 41]. The orthologs of bZIP63 were found in several plants regulating plant metabolism to pathogen defense [53, 54], and may participate in the signal crosstalk of ABA and auxin [41, 55]. ERD15 was identified as a central component in abiotic and biotic stress response in *Arabidopsis thaliana* [56]. Since the resources are limited, plants need to restrict their growth to ensure the defense for survival. Among the DNB genes, MYB3R5, TOPP9 and CDPK2 have been proved to be important negative regulators of plant growth. MYB3R5 inhibits plant growth by restricting the expression of the genes with mitotic functions [44]. While TOPP9 acts as a nucleocytoplasmic negative regulator of tip growth and affects the MAPKs-mediated defense [44].

Besides, several genes including AMT2, SYP121, CPK9, AtLEA5, AGB1 and SNAP33 in DNB members are not only involved in the ‘jasmonic acid(JA)-mediated signaling’ pathway, but also involved in other five pathways, i.e. ‘salicylic acid mediated signaling pathway’, ‘response to the abscisic acid stimulus’, ‘signal transduction by protein phosphorylation’, ‘regulation of innate immune response’, and ‘defense response to fungus’. These genes may mediate phytohormone signaling crosstalk to regulate plant defense [3, 24, 57]. The function enrichment analysis revealed that DNB genes were widely involved in plant response to environment and plant signaling hormones, such as ‘response to chitin’, ‘response to other organonitrogen compounds’, ‘regulation of innate immune response’, ‘abscisic acid-activated signaling pathway’, etc. These results strongly supported that the DNB genes play an important role in plant growth-to-defense transition.

Furthermore, we analyzed the network of DNB genes and highlighted many hub genes including SYP121, SYP122, WRKY33, AT5G2290, MPK11, etc. These hub genes may perform key functions to plant growth defense. The top 1 hub gene SYP121 has been proved to promiscuously form SDS-resistant SNARE (soluble *N*-ethylmaleimide–sensitive factor protein attachment protein receptor) core complex together with the SNAP33 adaptor and a subset of VAMPs. Through the core complex, SYP121 drives vesicle traffic at the plasma membrane of cells throughout the vegetative plant [58, 59]. SYP121 plays a central role in plant growth and defense for that it not only facilitates targeted vesicle traffic for defense against pathogen attack to coordinate systemic immunity [20, 60], but also participates in ABA pathway of cellular responses to drought and water stress [59, 61]. The study of exogenous applications for labeled JAs indicated that JAs were able to be translocated to distal sites as signals [62, 63]. Some studies suggest that JAs work together with system in the same signaling pathway that regulates systemic defense response with the mediation of SYP121 [64, 65]. The top 2nd hub gene SYP122 (up-regulated) shares partial functional redundancy in vivo with the close homolog SYP121 with similar structures [66, 67].

In a previous research, the dynamic transcriptional regulation mechanism of JA gene regulatory network was studied [51]. The results showed that MeJA induces a burst of transcriptional activities which generate diverse expression patterns over time. In this study, we focus on the defense responses of plants induced by MeJA. With our special data analysis, we detected the tipping point of the growth-to-defense transition and identified a group of genes closely related to plant defense. The identified genes were proved to play a key role in plant defense and negative regulation of plant growth by previous studies [44, 68]. The top genes identified were involved in regulating the redistribution of metabolism, plant responding to stress, and defensive signals transport. These identified genes provide candidates for engineering breeding that improves crop resistance or minimizes the consumption of resources to increase yields.

The DNB method can identify the critical transition points by analyzing the drastic fluctuations of a closely interacted molecular module in a system based on the high-throughput time-series data. Except for plant growth-defense transition, the DNB method can implement other situations such as disease or insect attacks if the perturbation data satisfying three criteria are available. At the situation of disease attacks, the DNB method has been proved to be effective for identifying the transition point of the disease during its progression [37]. To check whether MeJA-induced transition points are same every time, there should be some same experimental dataset for the analysis. If different systems are used for the comparison, the transition points of the systems can be identified but the key genes involved in the biological process are different because plants use different strategies to defense against different attacks [69].

In this work, we provide a new perspective for understanding the molecular mechanisms of growth-to-defense transition. With a dynamic network analysis of MeJA-induced plant resistance, the tipping point of plant defense was detected. At the tipping point, the JA response genes in format of a module fluctuated drastically in expression. And a group of members were identified as the dynamic network biomarkers for this critical transition. The analysis showed that some of the identified genes have been widely proved by previous studies to play roles in plant defense and negative regulation in plant growth. Besides, we also provide a database with the key genes of plant defense induced by MeJA.

## Methods

### Data collection and pre-processing

The raw reads of high-resolution time-series RNA-seq dataset are accessible by number PRJNA224133 in the NCBI (National Center for Biotechnology Information) SRA (Short Read Archive) database (http://www.ncbi.nlm.nih.gov/sra/). The *Arabidopsis* was treated by MeJA as case and treated by mock as control. For each of the 13 time points (0.25 h, 0.5 h, 1 h, 1.5 h, 2 h, 3 h, 4 h, 5 h, 6 h, 7 h, 8 h, 9 h, 10 h, 12 h) over a 12-hour period, 4 biological replicates and 4 technical replicates were performed. More details of the experiment can be found in the original report [70]. The raw reads were assessed with FastQC (v0.11.6; https://www.bioinformatics.babraham.ac.uk /projects/fastqc/) for quality control. HISAT (V2.1.0) was used to alignment the reads to *the Arabidopsis thaliana* reference genome(TAIR10) [71]. After alignment, StringTie (V1.3.3b) was used to assemble the aligned reads into full transcripts and to quantify Transcripts Per Kilobase of exon model per Million mapped reads (TPM) values of all genes. The gene expression level in each sample was calculated from the median value of four technical replicates for this sample. For further analysis, the genes with low abundance (TPM < 1) were filtered out. The filtered gene expression data was listed in Supplementary Table S7.

### Differential expression analysis

Differential gene expression analysis was performed using the R packge EBSeq (V0.36) which was developed based on an empirical Bayesian hierarchical model [72]. The genes with fold change (FC) > 1.5 and false discovery rate (FDR) < 0.05 were selected as DEGs.

### Function enrichment and visualization

The GO and KEGG enrichment analysis for DNB genes were performed using R package topGO (V2.38.1, https://rdrr.io/bioc/topGO/) which was based on a hypergeometric test. The *Arabidopsis* GO (V3.10.0, https://bioconductor.org/packages/org.At.tair.db/) was chosen for the annotation analysis. The significant enrichment GO terms were chosen by the adjusted *P*-value < 0.05. The dotplot and barplot were used for the visualization of the enriched GO terms.

### STRING network analysis

To further analyze the function of DNB genes, the network between DNB and DEG genes was constructed with the STRING database as the background interactions. The parameter of comprehensive score was set to 0.6 for the filter of the network [73]. In the STRING network, the node represented the protein produced by a single and protein-coding gene locus and the edge represented protein-protein interactions (PPIs) including known and predicted interactions. The PPI score in the dataset indicated the confidence of interaction.

### Network visualization

Cytoscape3.71 was used to visualize the network of gene interactions [74]. To survey the potential hub genes in growth-defense trends-off, the CytoHubba plug-in in Cytoscape was used to identify the hub genes.

## Supplementary Information

**Additional file 1 **: **Supplementary Material.** Figures for DNB module and CI index.

**Additional file 2 **: **Supplementary Table S1.** List of DNB genes and their functional descriptions.

**Additional file 3 **: **Supplementary Table S2.** Functional enrichment for DNB genes.

**Additional file 4 **: **Supplementary Table S3.** List of DNB genes assigned KEGG pathways.

**Additional file 5 **: **Supplementary Table S4.** List of differentially expressed genes between the defense tipping point and previous time point.

**Additional file 6 **: **Supplementary Table S5.** The network of DNB genes and DEGs.

**Additional file 7 **: **Supplementary Table S6.** List of the top 10 hub genes of the core network of DNB genes and DEGs.

**Additional file 8 **: **Supplementary Table S7.** The list of filtered gene expression data.

## Data Availability

The raw RNA-seq data analyzed in this study are available in the NCBI SRA database, https://www.ncbi.nlm.nih.gov/sra/PRJNA224133 (accession BioProject number: PRJNA224133). The data supporting the findings of this study were included in the supplementary files.

## Abbreviations

MeJA: Methyl jasmonate.
DNB: Dynamic network biomarker.
JA: Jasmonic acid.
JAZ: Jasmonate zim domain.
WGCNA: Weighted correlation network analysis.
SD: Standard deviation.
PCC: Pearson correlation coefficient.
CI: Composite index.
PPI: Protein-protein interactions.
DEG: Differentially expressed genes.
GO: Gene Ontology.
KEGG: Kyoto Encyclopedia of Genes and Genomes.
TPM: Per million mapped reads.
FC: Fold change.
FDR: False discovery rate.
