# Supplementary material for: Genome-wide dynamic network analysis reveals the potential genes for MeJA-induced growth-to-defense transition
Source: BMC Plant Biol. 2021 Oct 6;21:450. doi: 10.1186/s12870-021-03185-1 (PMC8493714; doi:10.1186/s12870-021-03185-1)
Supplement: Supplementary file 1 — Additional file 1 : Supplementary Material. Figures for DNB module and CI index. [file 12870_2021_3185_MOESM1_ESM.doc]

## Supplementary Material

## Genome-wide dynamic network analysis reveals the potential genes for MeJA-induced growth-to-defense transition

Tengfei Wang^1,2,3^ and Xiujun Zhang^1,2^,*

^1^ CAS Key Laboratory of Plant Germplasm Enhancement and Specialty Agriculture, Wuhan Botanical Garden, Chinese Academy of Sciences, Wuhan, 430074, China

^2^ Center of Economic Botany, Core Botanical Gardens, Chinese Academy of Sciences, Wuhan, 430074, China

^3^ University of Chinese Academy of Sciences, Beijing, 100049, China

* To whom correspondence should be addressed to [zhangxj@wbgcas.cn](mailto:zhangxj@wbgcas.cn).


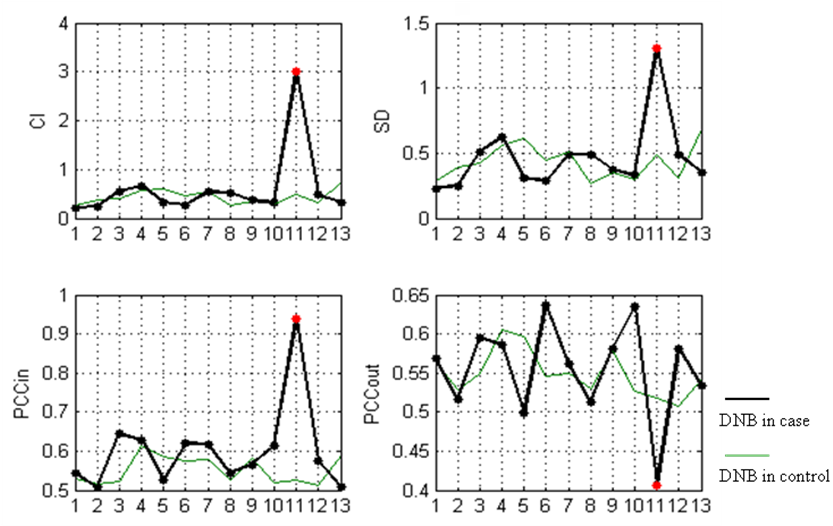


**Figure S1** The CI, SD, PCCin and PCCout of DNB module. The CI, SD, PCCin and PCCout of DNB module were marked in black in data from case samples (MeJA treated) and were marked in green in data from control samples (MOCK treated). The red dots represent the tipping point of transition identified by DNB.


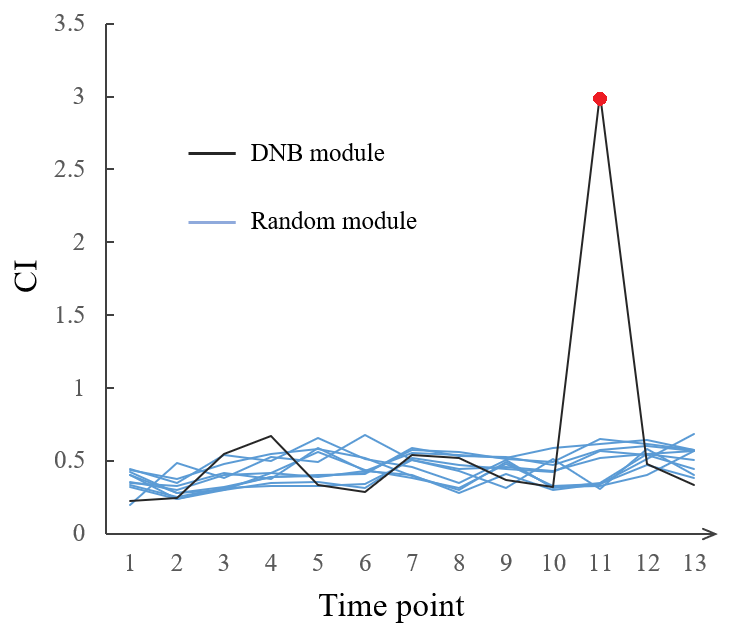


**Figure S2.** The CI of DNB module and random modules in different periods. The CI of DNB module was marked in black and random module were marked in green. The highest CI score for DNB module marked as a red dot at point 11.
